# Supplementary material for: HLAIIPred: cross-attention mechanism for modeling the interaction of HLA class II molecules with peptides
Source: Commun Biol. 2025 Jul 30;8:1133. doi: 10.1038/s42003-025-08500-2 (PMC12310933; doi:10.1038/s42003-025-08500-2)
Supplement: Supplementary file 1 — Supplementary information [file 42003_2025_8500_MOESM1_ESM.pdf]

Supplementary Information

HLAIIPred: Cross-Attention Mechanism for Modeling the Interaction of HLA Class II Molecules with Peptides

Mojtaba Haghighatlari[1*], Nicholas Marze[2], Robert Seward[3], Andrew Ciarla[3], Rachel Hindin[3], Jennifer Calderini[3], Benjamin Keenan[3], Santosh Dhule[3], Sarah Hall-Swan[1], Timothy P. Hickling[2], Eric Bennett[2], Brajesh Rai[1], Sophie Tourdot[3]

[1]Machine Learning and Computational Sciences, Pfizer Research and Development, Cambridge, MA, USA
[2]Biomedicine Design, Pfizer Research and Development, Cambridge, MA, USA
[3]Pharmacokinetics, Dynamics and Metabolism, Pfizer Research and Development, Andover, MA, USA
[*]corresponding author:mojtaba.haghighatlari@pfizer.com

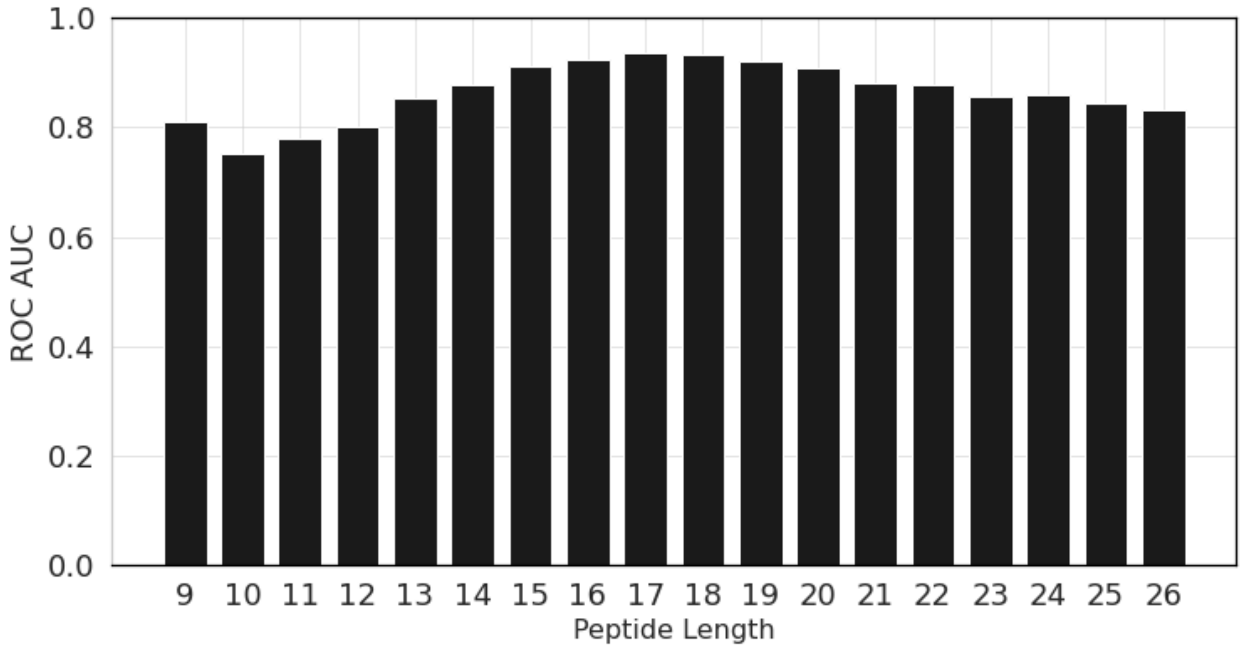

Supplementary Figure 1 depicts the performance of HLAIIPred in terms of ROC-AUC for different peptide lengths in the test set.

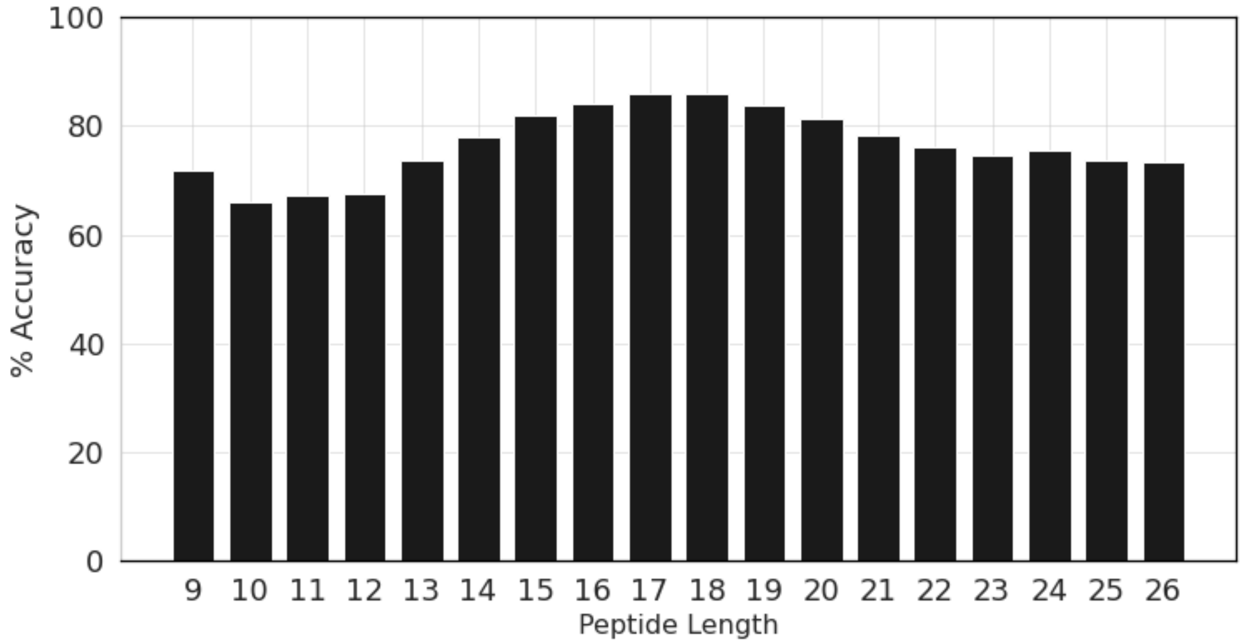

Supplementary Figure 2 depicts the performance of HLAIIPred in terms of Accuracy(%) for different peptide lengths in the test set.
