# Supplementary material for: HLAIIPred: cross-attention mechanism for modeling the interaction of HLA class II molecules with peptides
Source: Commun Biol. 2025 Jul 30;8:1133. doi: 10.1038/s42003-025-08500-2 (PMC12310933; doi:10.1038/s42003-025-08500-2)
Supplement: Supplementary file 3 — Description of Additional Supplementary Files [file 42003_2025_8500_MOESM3_ESM.pdf]

Description of Additional Supplementary Files

Supplementary data 1: The Bococizumab data behind Figure 5.

Supplementary Data 1-A: MAPPs-identified presented peptides from bococizumab heavy chain along with donor identification, mass spec peptide count, and germline or non-germline as binary values.

Supplementary Data 1-B: MAPPs-identified presented peptides from bococizumab light chain along with donor identification, mass spec peptide count, and germline or non-germline as binary values.

Supplementary Data 1-C: The donor allotypes
